# Current perspectives on risk prediction and genetic basis of Brugada syndrome

**DOI:** 10.3389/fcvm.2025.1722105

**Published:** 2025-12-12

**Authors:** Priya Bhardwaj, Dorte Stavnem, Stine Bøttcher Jacobsen, Bo Gregers Winkel, Jacob Tfelt-Hansen

**Affiliations:** 1 Department of Cardiology, The Heart Centre, Copenhagen University Hospital, Rigshospitalet, Copenhagen, Denmark; 2 Section of Forensic Genetics, Department of Forensic Medicine, University of Copenhagen, Copenhagen, Denmark

**Keywords:** arrhythmia, arrhythmic syncope, atrial fibrillation, Brugada syndrome, *BRUGADA-RISK* model, electrocardiographic pattern, *SCN5A*, *Shanghai score*

## Abstract

Brugada syndrome (BrS) is an inherited arrhythmia disorder and a major cause of sudden cardiac death below 50 years. Despite more than three decades of research, diagnosis and risk prediction remain challenging due to variable presentation and incomplete understanding of its genetic basis. The Brugada electrocardiographic pattern is central to diagnosis but lacks specificity, while different scoring systems offer structured assessment yet perform inconsistently in asymptomatic or intermediate-risk patients. *SCN5A* is the only gene with definitive evidence for causality, but incomplete penetrance and polygenic effects limit its clinical utility. Important gaps remain, including the low diagnostic yield of genetic testing, the unclear course of asymptomatic BrS patients with spontaneous type I electrocardiographic pattern and in geno-negative BrS patients, and the limited validation of current risk models. In this mini review, we explore these challenges and discuss new directions, that could move the field toward more accurate and personalized management.

## Introduction

Brugada syndrome (BrS) is an inherited primary electrical disorder, characterized by ST-segment elevation and T-wave inversion in the right precordial leads, particularly V1 and V2 (1, 2). First described by the Brugada brothers in 1992, the syndrome was recognized in eight patients, including two siblings, who presented with recurrent aborted sudden death in the absence of structural heart disease, exhibiting a coved-type ST-segment elevation, right bundle branch block, and normal QT interval (3, 4). Since then, BrS has been established as a major cause of arrhythmic death in young, otherwise healthy individuals (5–7).

While most BrS patients remain asymptomatic, some may present with ventricular fibrillation (VF), arrhythmic syncope (typically at rest or during fever), or atrial fibrillation in structurally normal hearts (8). The condition predominantly affects males, with the first episode of VF typically occurring at a mean age of 41 ± 15 years (2). However, BrS is phenotypically dynamic, and subtle or concealed patterns can complicate diagnosis. Even when diagnosis is achieved, accurate risk stratification is a major challenge, yet critical for guiding management regarding lifestyle advice, medication, ablation and implantable cardioverter-defibrillator (ICD) therapy (9). Both clinical and genetic factors contribute to risk assessment, but the optimal integration of these determinants remains an active area of investigation.

This mini review provides a concise overview of current knowledge on BrS risk prediction, focusing on clinical stratification tools, genetic contributions, and highlighting novel approaches to support precision risk assessment and individualized management.

## Diagnostic evaluation and risk stratification systems in Brugada syndrome

Diagnosis of BrS requires a type 1 Brugada electrocardiographic (ECG) pattern, which may be spontaneous or transient. Other conditions that may mimic this, must be excluded. Pharmacological provocation with sodium channel blockers (SCB) or fever can unmask a type 1 pattern. An induced pattern is considered less specific, as it occurs in 2%–4% of healthy individuals and at higher prevalence in patients with other arrhythmia substrates (10). Pharmacological provocation remains central in uncovering concealed BrS, but the selection of who to test and its interpretation requires caution, and standardized protocols are still evolving (11). Therefore, confirmation of BrS requires accompanying clinical features, such as documented ventricular arrhythmia (VA), syncope, or a relevant family history (1).

Sudden cardiac arrest (SCA) may be the first manifestation of BrS. Systematic evaluation of the survivors and their relatives is essential to identify underlying inherited conditions. In a cohort of 155 nonischemic SCA survivors and 282 relatives, systematic evaluation identified an inherited cardiac disease in 49% of probands (including seven cases of BrS) and 15% of relatives (including one case of BrS type 1) (12). Most probands received ICDs, and relatives benefited from genetic counseling and early management. These findings emphasize the importance of systematic work-up for early diagnosis and targeted intervention.

**Figure 1:**
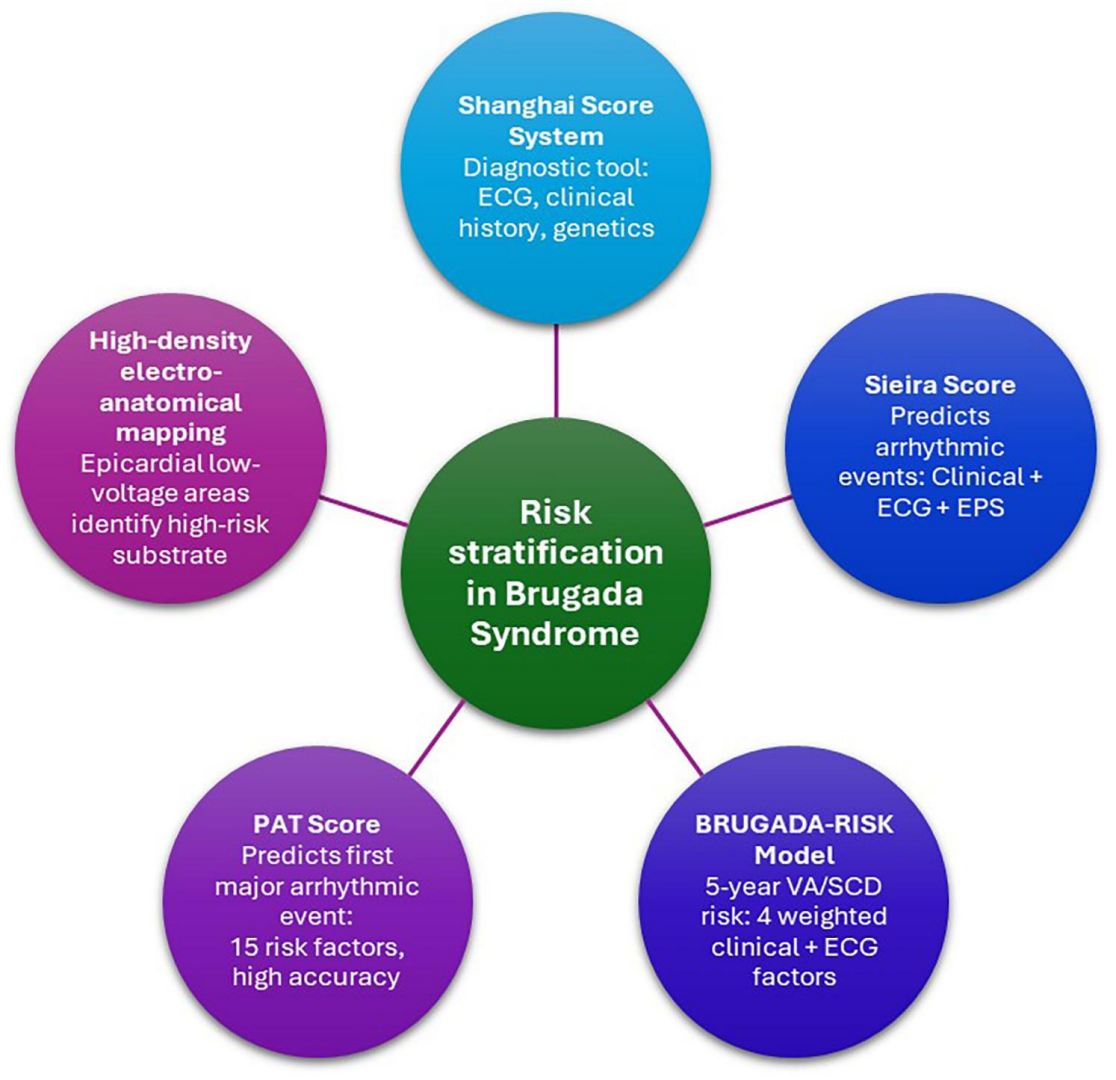
Overview of risk stratification models in Brugada syndrome. ECG, electrocardiogram; EPS, electrophysiological study; VA, ventricular arrhythmia; SCD, sudden cardiac death; PAT, Predicting Arrhythmic evenT.

Predicting future arrhythmic risk remains challenging, particularly in asymptomatic patients or those with borderline findings. To address this, several diagnostic and risk stratification scoring systems have been developed on identifying high-risk patients (13–17) (Figure 1).

The *Shanghai Score* was established in 2017 to address concerns about overdiagnosis, particularly due to false-positive SCB testing in healthy individuals (11, 12) This point-based system incorporates a positive history of 1) suspected arrhythmogenic syncope, 2) early onset of atrial fibrillation/flutter, 3) nocturnal agonal respiration, 4) documented VF/polymorphic ventricular tachycardia (VT), 5) positive genotype (*SCN5A*), 6) definite BrS in first- or second-degree family members and 7) family history of sudden cardiac death (SCD). Higher cumulative scores indicate probable or definite BrS. A validation study in ∼400 patients with suspected BrS (18) found no malignant VA among patients (∼12% of cohort) with a low score (categorized as either non-diagnostic or possible BrS). The *Shanghai Score* improves specificity, especially in cases of pharmacologically induced type 1 ECG, but requires careful documentation of phenotypic and family history details.

*Sieira score* was among the first, estimating event free survival at 1, 5 and 10 years following diagnosis (15), yet its predictive accuracy was limited (19–21). To improve this, the *BRUGADA-RISK* model was developed (22) based on a multicenter study of 1,100 patients with BrS, 906 of whom were asymptomatic. A total of 114 patients experienced primary endpoints of VA or SCD. VA was defined as aborted SCD or documented sustained VT/VF on monitoring or ICD interrogation. The model was validated using out-of-sample cross-validation. The study proposed four significant risk factors: probable arrhythmia-related syncope, or ECG changes including either spontaneous type 1 BrS morphology, the revelation of type 1 BrS pattern during elevated leads test, and presence of early repolarization pattern in the peripheral leads. Point values were weighted based on their individual predictive power. The cumulative score translates directly into a patient's estimated 5-year risk of VA or SCD. However, this model faced criticism for outcome bias by including appropriate ICD therapies in addition to SCD as primary endpoints, potentially overestimating SCD risk since not all ICD-terminated arrhythmias are fatal. Such limitations warrant careful consideration in clinical practice (21).

The latest advancement is the *Predicting Arrhythmic evenT (PAT) score*, derived from a worldwide pooled analysis of 7,358 patients (23). Fifteen of 23 identified risk factors were significant, and their pooled odds ratios formed the basis of the scoring system. Internal and external validation showed superior discrimination compared with *BRUGADA-RISK*, *Shanghai Score*, and *Sieira score*. A *PAT score* ≥10 predicted the first major arrhythmic event with 95.5% sensitivity and 89.1% specificity, making it a promising tool for primary prevention in BrS.

Beyond scoring systems, various diagnostic modalities have been evaluated for their ability to identify high-risk patients. *Delise et al*. proposed the use of electrophysiological studies (EPS) as part of a multiparametric approach (14). However, EPS and its diagnostic utility is considered controversial, especially among asymptomatic patients (10, 24, 25). This is supported by a systematic review by *Mascia et al*., who evaluated 1,318 patients with drug-induced type-1 pattern undergoing EPS. No significant difference in arrhythmic events was observed between EPS positive-, negative-, or non-studied groups over a mean follow-up of 5.1 years, suggesting that EPS may have limited prognostic value in this subgroup (26).

To refine substrate assessment, *Letsas et al*. (17) applied high-density electroanatomical mapping and identified epicardial abnormalities in the right ventricular outflow tract. Low-voltage areas effectively distinguished symptomatic from asymptomatic patients, indicating the value of this mapping technique for identifying high-risk patients.

Despite advances, stratifying SCD risk in asymptomatic and intermediate-risk individuals poses ongoing challenges (24). *Wilde* et al. (21) recently highlighted key methodological limitations: surrogate endpoints may overestimate actual risk, and most models are static, failing to account for changes in risk over time. The authors advocate for disease-specific surrogate endpoints and dynamic risk models that better reflect individual risk.

## The evolving genetics of Brugada syndrome

Although BrS is recognized as a heritable disorder, the diagnostic yield of genetic testing remains relatively low, limiting its utility in risk stratification and clinical decision-making.

Currently, a pathogenic or likely pathogenic variant is identified in approximately 20% of clinically diagnosed BrS patients (27, 28), leaving the majority genetically unexplained (29, 30). Historically, more than 20 genes were proposed to be associated with BrS. However, following the systematic re-evaluation of gene-disease-associations by the Clinical Genome Resource (31), only one gene, *SCN5A*, has been determined to have definite evidence for causality (32, 33). Other implicated genes have either been shown to be too prevalent in the general population to be causal or have insufficient evidence, and are therefore considered of disputed significance (4, 31).

*SCN5A* encodes the pore-forming α-subunit of Na_v_1.5, which is a voltage-gated sodium channel responsible for the inward sodium current in excitable cells (34). Loss-of-function (LOF) variants in *SCN5A* are the only established genetic mechanism of BrS, resulting in Na_v_1.5 channel dysfunction or reduced expression, producing the characteristic conduction abnormalities (35, 36). While inheritance patterns are often described as autosomal dominant, it has been shown that the genetic influence is more nuanced and with ethnic differences (37).

Notably, genotype-phenotype discrepancies are common. In families with *SCN5A* variants, individuals negative for the familial variant may still express the typical ECG phenotype, while some carriers remain lifelong asymptomatic (4, 38). *SCN5A* variants also overlap with other inherited cardiac diseases and syndromes, emphasizing the need to interpret genetic results within the context of the phenotype and family history (28, 36, 39). Despite these challenges, the presence of a pathogenic/likely pathogenic variant in *SCN5A* has prognostic implications: carriers tend to experience the first arrhythmic event at an earlier age than genotype-negative patients, and predicted LOF variants have been indicated to be a predictor of VA risk in BrS patients (27, 40–42). Mechanistic insights have further refined our understanding of how *SCN5A* variants influence phenotype expression: Using combined genetic and high-density epicardial mapping, *Ciconte* et al. (43) demonstrated that *SCN5A* variant carriers exhibit a larger epicardial arrhythmogenic substrate, prolonged electrograms, and higher prevalence of spontaneous type I ECG and VT/VF events than non-carriers. These findings provide strong evidence that *SCN5A* loss-of function is not only a molecular marker but also a determinant of structural electrical remodeling in BrS.

Recent studies indicate that BrS is often more complex than a simple monogenic disorder. The combination of incomplete penetrance, variable expressivity and genotype-negative phenotypes suggest a multifactorial, polygenic model, where both rare and common genetic variants contribute to genetic susceptibility. Genome-wide association studies, have identified several common variants at loci including *SCN10A* and *HEY2* that modulate BrS risk and may explain phenotypic variability (37, 44–47), but have not been shown to benefit the clinic care (48).

Clinical integration of genetic testing requires caution. Targeted genetic testing is recommended primarily for diagnosed patients, as it can inform cascade family screening and facilitate early identification of at-risk relatives (49). Both the 2011 and 2022 Expert Consensus documents advocate genetic counselling for probands and family members when a pathogenic/likely pathogenic variant is detected (50, 51).

## Discussion

### Integrating clinical, genetic, and electrophysiological perspectives

More than 30 years after its first description, BrS diagnosis and prognosis are still surrounded by controversy. Clinical presentation ranges from SCA to complete absence of symptoms. Genetics adds another layer of complexity. Some authors caution against overinterpreting genetic findings in risk stratification, particularly since many carriers remain asymptomatic, and many patients lack identifiable variants (30). Others see potential in refining genetic interpretation by focusing on variant location within *SCN5A* or integrating polygenic risk scores, which may provide a more nuanced estimate of arrhythmic risk (48). In this context, *Kukavica et al.* (52) recently demonstrated showed that male sex, the type of *SCN5A* variant, and a BrS-specific polygenic risk score are each associated with arrhythmic risk. Together, these factors form a nonmodifiable risk profile that helps identify patients at higher risk regardless of symptoms or ECG pattern.

The clinical implications of these mechanistic links were underscored by *Santinelli et al*. (53), who prospectively followed symptomatic BrS patients receiving ICDs with or without epicardial ablation. Substrate size, *SCN5A* status, and prior cardiac arrest independently predicted VF recurrence. Epicardial ablation combined with ICD implantation reduced arrhythmic events over long-term follow-up. Together, these findings bridge the gap between genetics and therapy, suggesting that *SCN5A* variants might identify patients with a more extensive substrate who may derive particular benefit from epicardial ablation.

### Limitations of current risk stratification

Risk stratification in BrS relies on multiparametric tools including clinical risk scores, EPS and emerging procedural assessments (54, 55). Current risk models often include small cohorts, heterogeneous inclusion criteria, and limited validation, making the prediction of arrhythmic events challenging (54, 55). The type 1 ECG pattern, although diagnostic, can fluctuate due to lead placement, autonomic tone, fever or drug provocation. Lead placement and SCB dosing protocols currently vary across centers, and the latest consensus could not issue unified recommendations due to limited evidence. Other ECG markers, such as the aVR sign or fragmented QRS complexes, have been suggested to identify higher-risk patients, but results are inconsistent across studies and populations, leaving some uncertainty in interpretation (56).

The natural history of phenotype positive-genotype negative individuals is poorly understood, complicating recommendations for monitoring, lifestyle modifications, or preventive interventions (4, 29). Many centers lack multidisciplinary teams to provide comprehensive genetic counseling, including discussions of penetrance, inheritance, and individualized risk (30, 57). In sports cardiology, determining eligibility is particularly tricky, as most BrS individuals may safely compete, but only after careful evaluation (58).

### Future directions

**Figure 2:**
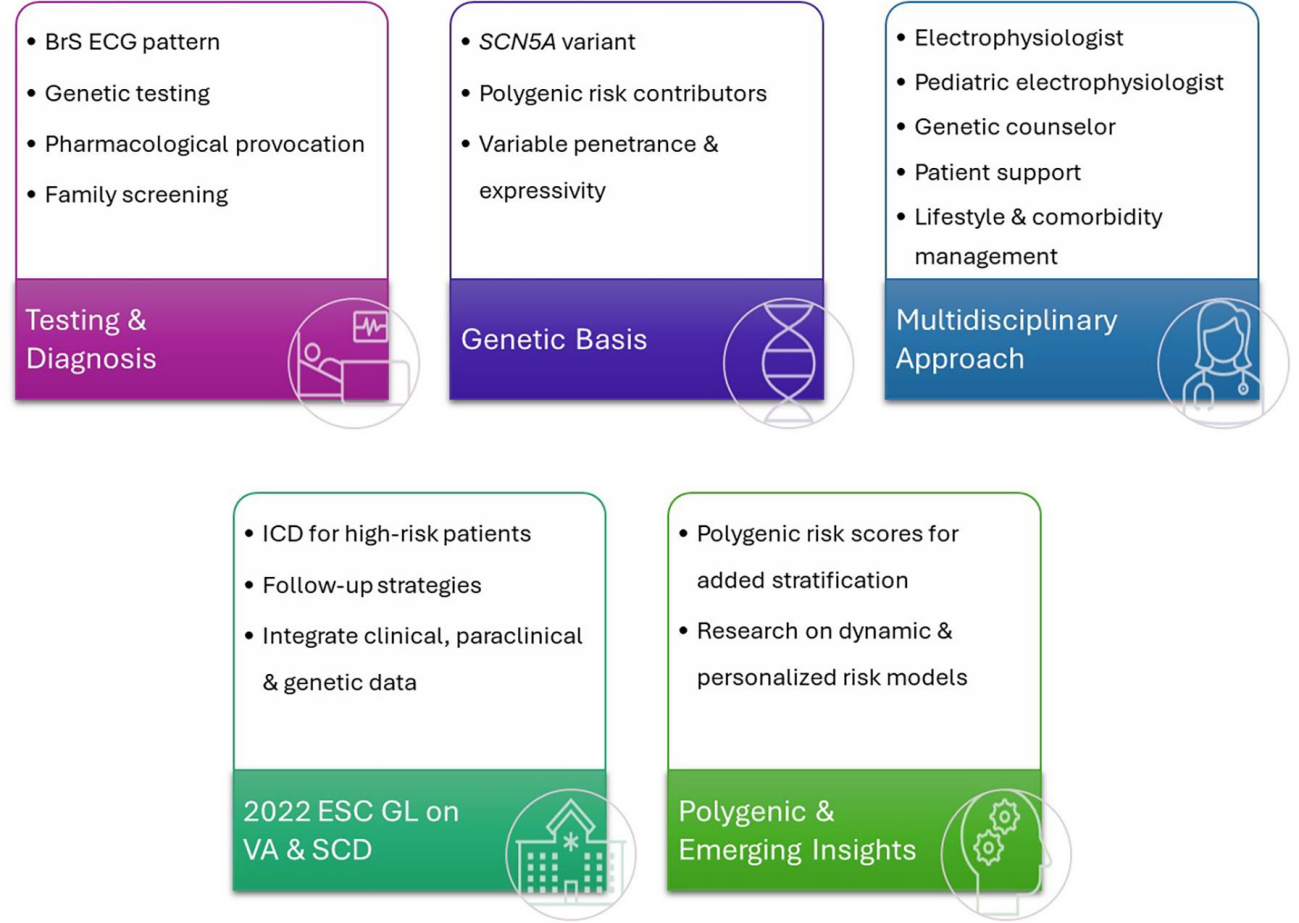
Comprehensive approach to Brugada syndrome. BrS, Brugada syndrome; ECG, electro; SCN5A, gene; VA, ventricular arrhythmia; SCD, sudden cardiac death; ICD, implantable cardioverter-defibrillator; ESC, European Society of Cardiology; GL, guidelines.

Future management strategies are likely to be increasingly personalized (Figure 2). Emerging research should focus on exploring ways to directly target the underlying genetic defects. Using advanced genetic tools such as *CRISPR* or allele-specific silencing, delivered through heart-targeted viral vectors, may offer a transition from symptomatic management toward potentially curative strategies (57, 59). The selection of BrS patients for gene treatment and when to initiate the gene treatment remains elusive.

Artificial Intelligence (AI) is another promising avenue. Early studies show how AI can reliably detect BrS patterns, raising the possibility of non-invasive screening and early risk identification (60). At a population level, large genomic datasets combined with AI and polygenic modeling could support predictive testing for SCD-associated *SCN5A* variants, enabling preventive strategies before symptoms appear, though careful ethical and familial considerations remain essential (57).

Finally, procedural approaches, such as right ventricular outflow tract epicardial ablation, may eventually provide alternatives to ICD implantation in select high-risk patients, particularly when devices carry added risk (61).

## Limitations of this review

While this mini review summarizes recent advances in BrS risk prediction and genetics, it is limited by the selective focus. Some studies discussed are based on heterogeneous patient populations or surrogate outcomes, which may restrict generalizability.

## Conclusion

BrS is moving toward a personalized, precision-medicine approach, integrating the clinical scenario, genetics, advanced ECG analytics, and targeted therapies. While controversies and gaps persist, these emerging tools offer real potential to improve early detection, refine risk stratification, and optimize management and ultimately reduce the burden of SCD while minimizing unnecessary interventions.
